# The efficacy of liposomal bupivacaine compared with traditional peri-articular injection for pain control following total knee arthroplasty: an updated meta-analysis of randomized controlled trials

**DOI:** 10.1186/s12891-019-2660-7

**Published:** 2019-06-29

**Authors:** Yuan Liu, Yi Zeng, Junfeng Zeng, Mingyang Li, Wenxing Wei, Bin Shen

**Affiliations:** Department of Orthopedics, West China Hospital, West China Medical School, Sichuan University, 37# Guoxue Road, Chengdu, 610041 Sichuan Province People’s Republic of China

**Keywords:** Total knee arthroplasty, Liposomal bupivacaine, Traditional peri-articular injection, Pain control, Randomized controlled trial, Meta-analysis

## Abstract

**Background:**

The efficacy of postoperative pain management is an important factor that influences the final outcome of total knee arthroplasty (TKA). Whether liposomal bupivacaine offers better efficacy compared with traditional peri-articular injection after TKA remains inconclusive. We conduct this study to compare the true efficacy of liposomal bupivacaine (LB) with traditional peri-articular injection (TPAI) following TKA.

**Materials and methods:**

Randomized controlled trials (RCTs) from PubMed, EMBASE, and the Cochrane Central Register of Controlled Trials (CENTRAL) and Web of Science were searched. Thirteen RCTs involving 1373 patients were finally included in our meta-analysis (LB = 691, TPAI = 682). The continuous and dichotomous outcome were collected in a standard form, and the data were analysed by using Review Manager 5.3 software. Finally, the results were presented in the forest plots.

**Result:**

The pooled data demonstrated that the postoperative visual analogue score (VAS) in the LB group was not significantly different compared with that in the TPAI group at every time period after TKA. The liposomal bupivacaine group had significantly lower consumption of morphine equivalents 24 to 72 h postoperatively and reduced incidence of nausea and vomiting after TKA compared with the TPAI group. Finally, the length of hospital stay in the two groups was not significantly different.

**Conclusion:**

Liposomal bupivacaine did not yield different results on the visual analogue scale compared with traditional peri-articular injection after total knee arthroplasty. However, liposomal bupivacaine was preferred in terms of lower consumption of morphine equivalents 24–72 h postoperatively and lower incidence of nausea and vomiting after total knee arthroplasty.

## Introduction

As the most effective treatment for advanced osteoarthritis, more than half of patients receive benefits from total knee arthroplasty (TKA), such as alleviation the pain, functional recovery of the knee joint and improvement of the quality of life. However, a limitation that cannot be ignored is that most patients still experience postoperative pain at different levels [1, 2]. Both surgeons and patients are very worried about this pain. On the one hand, inadequate pain control impedes physical exercise after TKA and increases the demand of rescued opioids. The former could influence the functional recovery and delay early rehabilitation, and the latter could be associated with more adverse effects, such as vomiting, nausea, dizziness, constipation, urinary retention and even respiratory depression [3, 4]. On the other hand, the efficacy of postoperative pain control could influence the patients’ satisfaction for the TKA procedure, and some patients do not undergo TKA due to unaccepted postoperative pain [5]. As a result, many types of postoperative pain control methods have been developed and applied.

Liposomal bupivacaine (LB; EXPAREL®, bupivacaine liposome injectable suspension; Pacira Pharmaceuticals, Inc., Parsippany, NJ) has a significant advantage of prolonging the effective time of bupivacaine to 72 h [6]. With the approval of the Food and Drug Administration (FDA) in 2103, liposomal bupivacaine has been safely and effectively applied to surgeries, including TKA, augmentation and mammaplasty, mastectomy with tissue expander placement [7]. To explore the efficacy of liposomal bupivacaine after total knee arthroplasty, a number of clinical trials have been performed to compare the LB with other methods, including peripheral nerve block and, traditional peri-articular injection (TPAI). TPAI is the best candidate to compare with liposomal bupivacaine in terms of minimal confounding bias [8] given that, liposomal bupivacaine was injected into the surroundings of the surgical site to control pain, which is consistent with TPAI.

According to our search results, 4 meta-analyses, 13 randomized controlled trials (RCTs), and 11 non-RCTs comparing liposomal bupivacaine with traditional peri-articular injection after TKA were identified. However, meta-analysis outcomes still need to be improved. Wang et al. [9] performed a meta-analysis with 3 RCTs and 2 non-RCTs comparing the LB with bupivacaine after TKA, but the control groups were mixed with local injection and the femoral nerve block, which could produce confounding bias. Kuang et al. [10] conduct a meta-analysis with 4 RCTs and 7 non-RCTs comparing the LB with traditional peri-articular injection after TKA, but the number of RCTs are limited. Sun et al. [11] perform a meta-analysis with 9 RCTs and 7 non-RCTs comparing the LB with traditional peri-articular injection after TKA. The inclusion of non-RCTs is unnecessary when the number of RCTs is sufficient. Therefore, we included 13 RCTs to perform an updated meta-analysis to obtain more believable outcomes to help clinical surgeons make a decision.

## Materials and methods

This meta-analysis was completed in accordance with the Preferred Reporting Items for Systematic Reviews and Meta-Analyses (PRISMA) guidelines for meta-analysis.

### Searching

Liposomal bupivacaine relevant studies from several electronic databases, including PubMed (1966 to Dec 2018), EMBASE (1980 to Dec 2018), and the Cochrane Central Register of Controlled Trials (CENTRAL, Dec 2018) and Web of Science (1966 to Dec 2018) were systematically searched by two reviewers. “Total knee arthroplasty OR replacement” and, “liposomal bupivacaine OR exparel” were used as search key words in connection with AND or OR. There was no limitation on language and locality.

### Inclusion criteria

Studies were selected if they met the following criteria in PICOS order: (1) Population: patients experiencing TKA who were demographically alike; (2) Intervention: peri-articular injection of liposomal bupivacaine; (3) Control intervention: traditional peri-articular injection including bupivacaine and cocktail (ropivacaine, epinephrine, ketorolac, clonidine, etc.); (4) Outcomes: postoperative pain score, morphine consumption, adverse effects and the length of stay; (5) Study design: randomized controlled trial (RCT).

### Data screening

Two reviewers independently screened the information listed in a standard form designed to screen the correlative data from included studies. The data extracted included authors, the year of publication, sample capacity, demographical information (age, gender, and body mass index), anaesthetic methods, the composition of agents by peri-articular injection in the experimental and control groups, follow-up, and power analysis. In particular, when we found two studies [12, 13] that reported the outcome by the box plot, we obtained the relevant literature and used the scientific method to obtain the mean and variance [14]. Any disagreements were unified through discussion. The primary outcome was postoperative VAS. Secondary outcomes included the consumption of morphine equivalents, the incidence of adverse effects such as nausea and vomiting (NAVO), and length of hospital stay (LOS).

### Risk of bias assessment

On the basis of the Cochrane Handbook for Systematic Reviews of Interventions 5.0, two reviewers respectively evaluate the methodological quality of included studies. We evaluated the RCTs using the “Cochrane collaboration’s tool for assessing the risk of bias,” which included the following key points: random sequence generation (selection bias); allocation concealment (selection bias); blinding of participants and personnel (performance bias); blinding of outcome assessment; incomplete outcome data (attrition bias); selective reporting (reporting bias); and other bias. A unified consensus was obtained if there were any different opinions.

### Evidence assessment with GRADE approach

The evidence assessment was determined using the guidelines of the grading of recommendations, assessment, development, and evaluation (GRADE) working group [15, 16]. The GRADE system uses a sequential assessment of the evidence quality and the evidence grades are divided into the following levels: (1) high, which indicates that further research is unlikely to alter confidence in the effect estimate; (2) moderate, which indicates that further research is likely to significantly alter confidence in the effect estimate and may change the estimate; (3) low, which indicates that further research is likely to significantly alter confidence in the effect estimate and to change the estimate; and (4) very low, which indicates that any effect estimate is uncertain. Uniformity of the estimated effects across studies and the extent to which the patients, interventions, and outcome measures are similar to those of interest may reduce or increase the evidence grade. As recommended by the GRADE working group, the lowest evidence quality for any of the outcomes was used to rate the overall evidence quality. The evidence quality was graded using GRADEpro online software (https://gradepro.org/).

### Statistical analysis

We used Review Manager 5.3 software to analyse pooled data. The effect value of mean differences (MDs) was used to weigh the effect size for continuous outcome. The effect value of relative risks (RRs) was used to measure the effect size for dichotomous outcome. We considered the result to be significantly different when a two-sided *p*-value < 0.05. We use the I^2^ statistic to test heterogeneity across the studies. We regarded a p-value ≤0.1 or an I^2^ > 50% as proof of heterogeneity. A random-effects model was used to eliminate the effect caused by high heterogeneity, and a fixed-effects model was adopted when the heterogeneity lacked statistical evidence. We performed subgroup analysis for postoperative VAS based on the cocktail and standard bupivacaine group to reduce the risk of bias.

## Results

### Search result

**Fig. 1:**
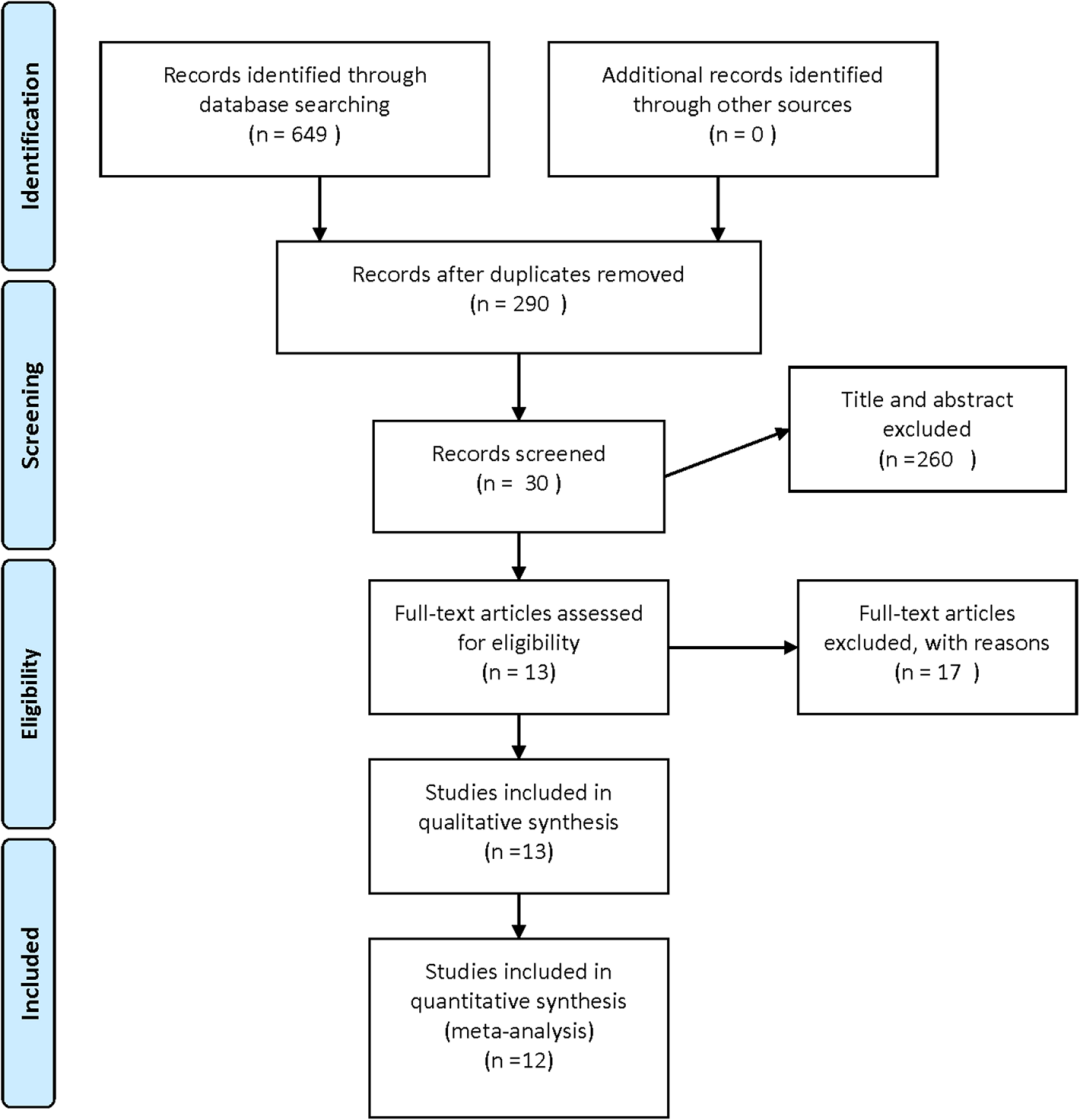
Flow of search results and selection procedure

A total of six-hundred-forty-nine relevant articles from electronic databases were identified depending on the search strategy. Three-hundred-fifty-nine duplicates were removed. Two-hundred-sixty studies were excluded after reading the abstract. After reading the full text, only nineteen studies comparing liposomal bupivacaine with traditional peri-articular injection were selected. Finally, according to the inclusion criteria for RCTs, 13 RCTs [12, 13, 17–27] with 1373 patients comparing liposomal bupivacaine with traditional peri-articular injection were included. (Fig. 1).

### Studies characteristics

Among of 13 RCTs, seven RCTs [13,17-18,22- 24,27] used bupivacaine as the control group, and the other six RCTs used the cocktail as the control group. The baseline characteristics of each study are presented in Table 1. All of 13 RCTs are published in the USA. Five [13, 19, 24, 25, 27] of 13 RCTs did not perform power analysis to estimate the sample size needed to acquire significant results. The length of follow-up varied from 0 to 8 weeks.

**Table 1:** The basic information of RCTs

		LB/traditional PAI								
studies	country	cases	age	female	BMI	AN	EG	control group	follow-up	PA
alijanipour 2016	USA	59/59	64.3/64.9	30/32	32.3/28.7	SA	LB266mg	bupivacaine	6 weeks	Y
Bramlett 2012	USA	25/34	61.1/62.2	12.0/23	31.2/31.5	GA	LB266mg	bupivacaine	36 days	Y
collis 2016	USA	54/51	63.7/63.5	29/37	34.1/35.7	GA	LB266mg	ropivacaine, epinephrine, ketorolac, clonidine	8 weeks	N
declaire 2017	USA	47/49	69.7/67.7	26/28	31.5/31.9	SA/GA	LB266mg	ropivacaine, ketorolac, morphine, epinephrine	NM	Y
Jain 2016	USA	63/62	68.3/67.5	44/45	33.3/33.3	SA	LB266mg	bupivacaine, epinephrine, morphine	NM	Y
mont 2017	USA	70/69	66/66	43/39	32.4/31.3	SA	LB266mg	bupivacaine	NM	Y
schroer 2015	USA	58/53	67/68.6	34/32	32/32	SA/GA	LB266mg	bupivacaine	3 weeks	Y
Schumer 2018	USA	66/64	NM	NM	NM	SA	LB266mg	bupivacaine	6 weeks	N
schwarzkopf 2016	USA	20/18	63/59	13/8.0	29.3/29.5	SA	LB266mg	ropivacaine, clonidine, Toradol, Epinepherine	NM	Y
smith 2017	USA	104/96	66/66	50/68	31.5/31.6	SA	LB266mg	bupivacaine	6 weeks	N
Snyder 2016	USA	35/35	67.3/65.6	13/20	30.68/31.29	SA/GA	LB266mg	ropivacaine,epinephrine morphine, ketorolac	10 days	N
suarez 2018	USA	52/52	68.1/67.3	33/26	30.8/32.01	SA	LB266mg	bupivacaine,lidocaine epinephrine, morphine, ketorolac	6 weeks	Y
zlotnicki 2018	USA	38/40	63.2/64.3	19/26	35.5/35.4	SA/GA	LB266mg	bupivacaine	NM	N

*AN* anesthesia, *SA* spinal anesthesia, *GA* general anesthesia, *NM* not mentioned, *PA* power analysis

### Risk of bias assessment

**Fig. 2:**
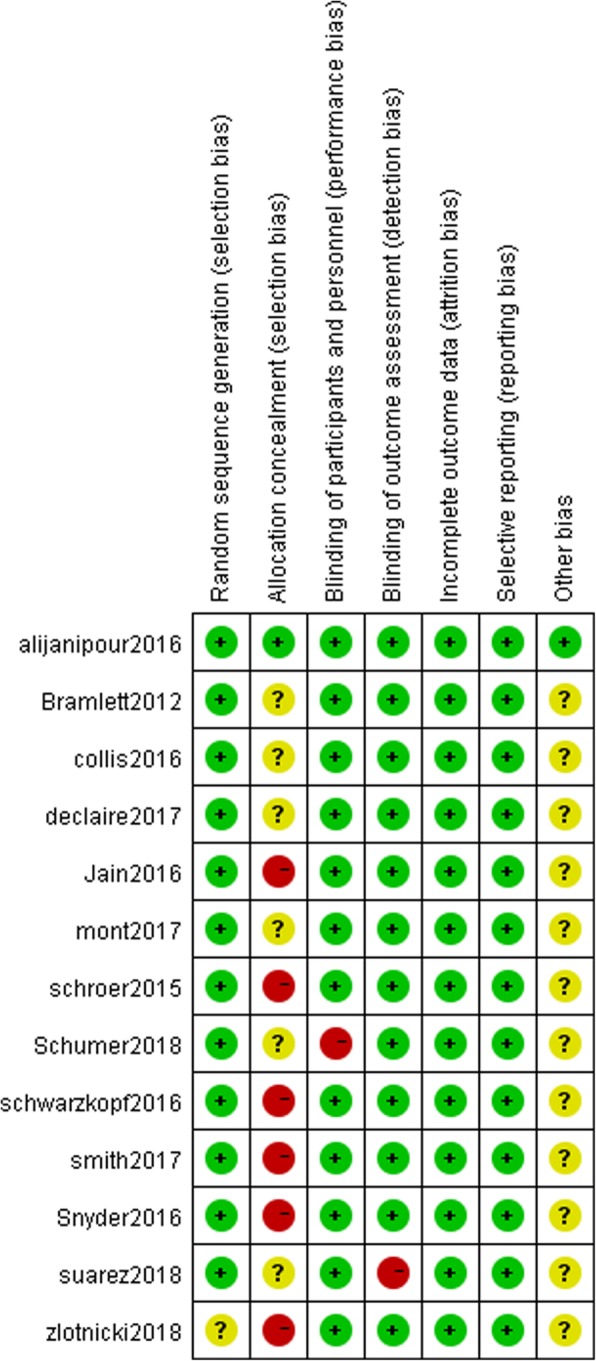
Methodological quality of included studies

**Fig. 3:**
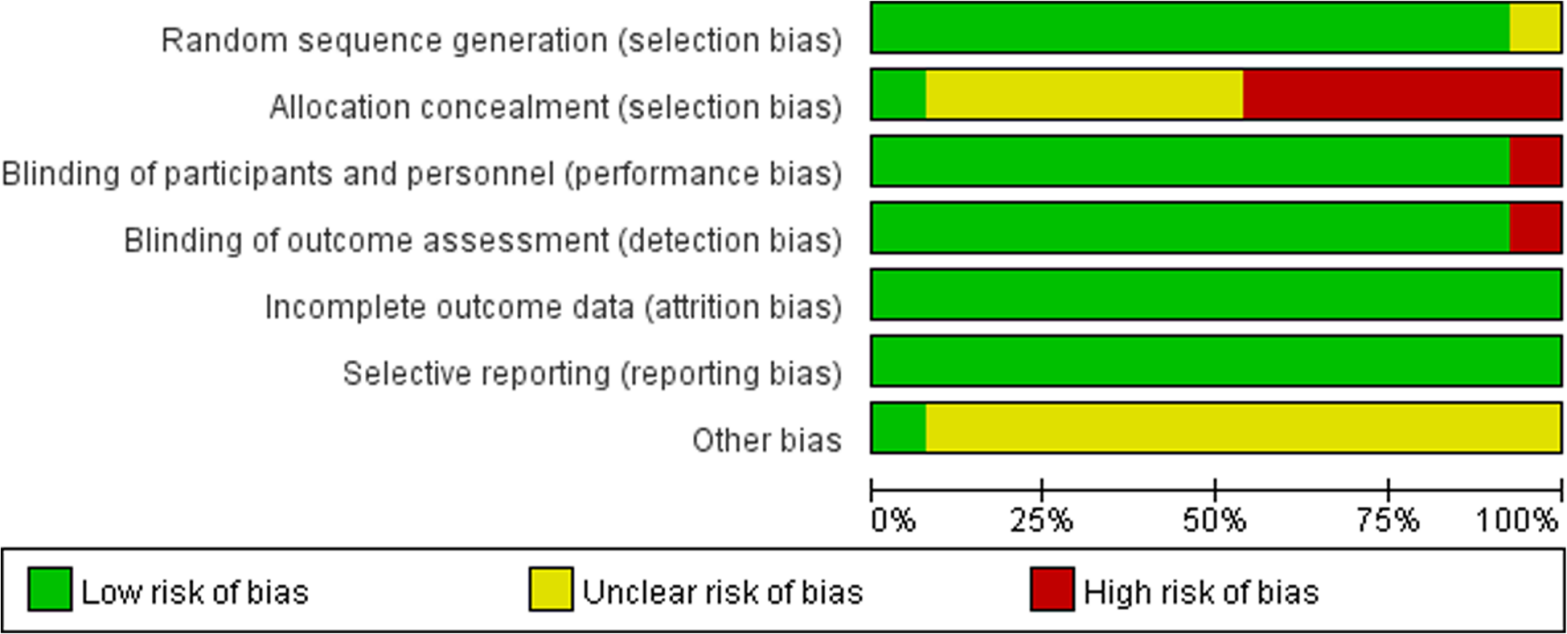
Risk of bias assessment of included studies

Methodological quality of 13 RCTs was evaluated with Cochrane collaboration’s tool for assessing the risk of bias [28]. Randomization was achieved using a random number table in 3 RCTs [13, 25, 26], excel software in 2 RCTs [17, 20], and a centralized randomization system in 2 RCTs [18, 22]. Only 2 RCTs [17, 19] described the concealment of allocation. Only 2 RCTs [24, 26] were single blinded, and the others were double-blinded. All RCTs mention information about withdrawal and dropout. The methodological quality of included studies was presented in Fig. 2. Judgements about each risk of bias item are presented as percentages across all included studies in Fig. 3.

### Primary outcome: the postoperative pain score

To decrease the bias caused by two different types of techniques in the control group, we divided the data into the bupivacaine and cocktail groups to perform a subgroup analysis regarding this outcome.

#### VAS during the first 24-h period after TKA

**Fig. 4:**
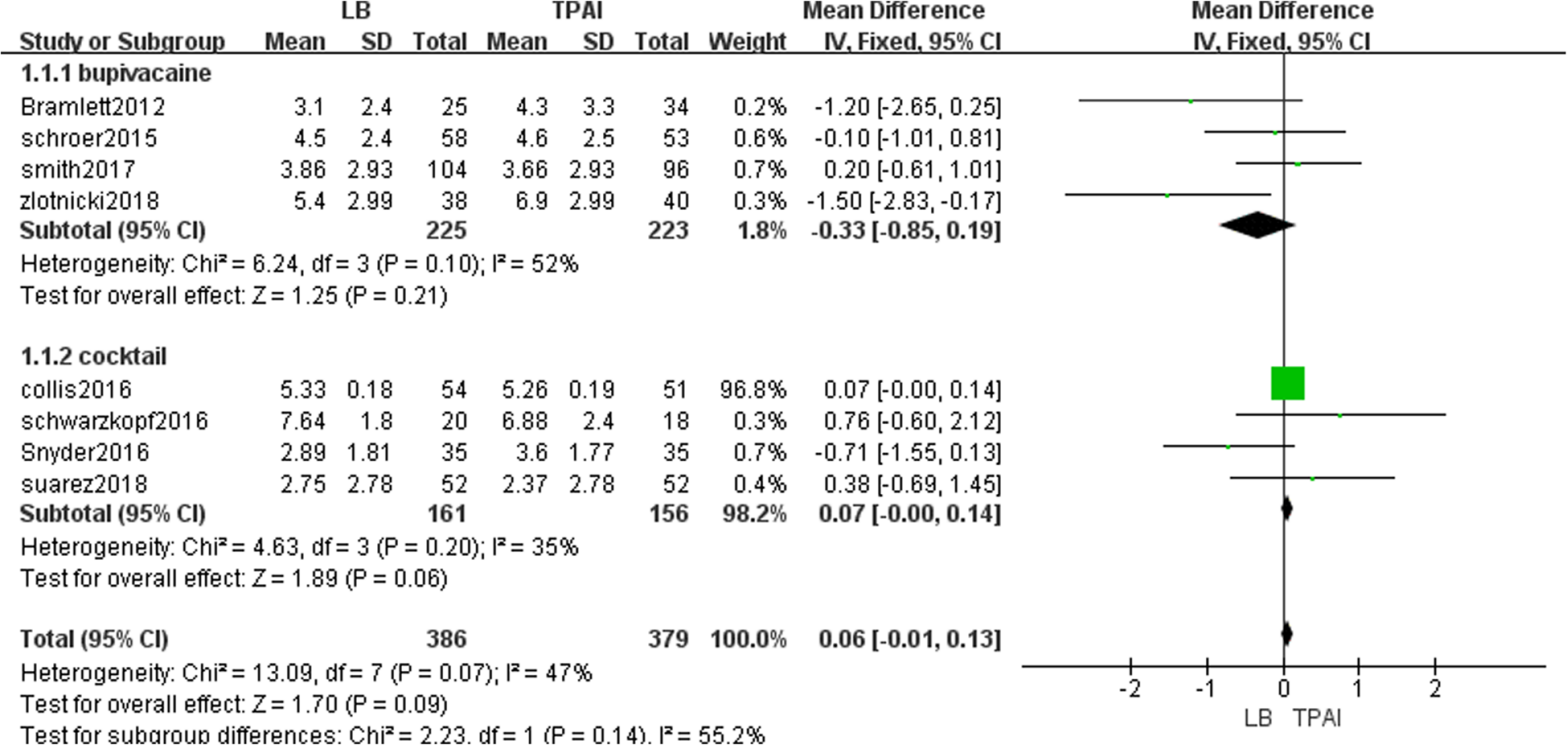
The frost plot of the VAS during the first 24 h after TKA

Eight studies involving 765 patients reported the VAS from 0 to 24 h postoperatively [12, 13, 18, 19, 23, 25–27]. The result showed that LB was not significantly different from TPAI regarding VAS during the first 24-h period after TKA (MD = 0.06, 95% CI: [− 0.01, 0.13], *P* = 0.09, I^2^ = 47%, Fig. 4). Fixed-effects meta-analysis revealed that the pain score in the liposomal bupivacaine group is not significantly lower than bupivacaine group (MD = -0.33, 95% CI: [− 0.85, 0.19], *P* = 0.21, I^2^ = 52%, Fig. 4), and not significantly higher than the cocktail group (MD = 0.77, 95% CI: [− 0.00, 0.14], *P* = 0.06, I^2^ = 35%, Fig. 4).

#### VAS during the second 24-h period after TKA

**Fig. 5:**
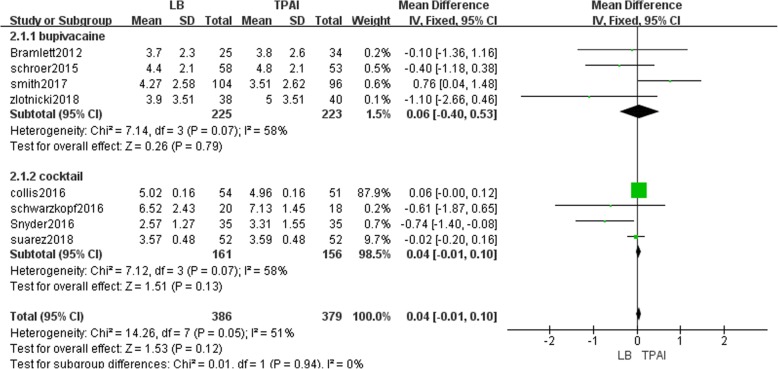
The frost plot of the VAS during the second 24 h after TKA

Eight studies involving 765 patients described the VAS from 24 to 48 h postoperatively [12, 13, 18, 19, 23, 25–27]. The result showed that LB was not significantly different from TPAI regarding VAS during the second 24-h period after TKA (MD = 0.04, 95% CI: [− 0.01, 0.10], *P* = 0.12, I^2^ = 51%, Fig. 5). Fixed-effects meta-analysis revealed that the pain score in the liposomal bupivacaine group is not significantly increased compared with the bupivacaine group (MD = -0.06, 95% CI: [− 0.40, 0.53], *P* = 0.79, I^2^ = 58%, Fig. 5), and not significantly increased compared with the cocktail group (MD = 0.04, 95% CI: [− 0.01, 0.10], *P* = 0.13, I^2^ = 58%, Fig. 5).

#### VAS during the third 24-h period after TKA

**Fig. 6:**
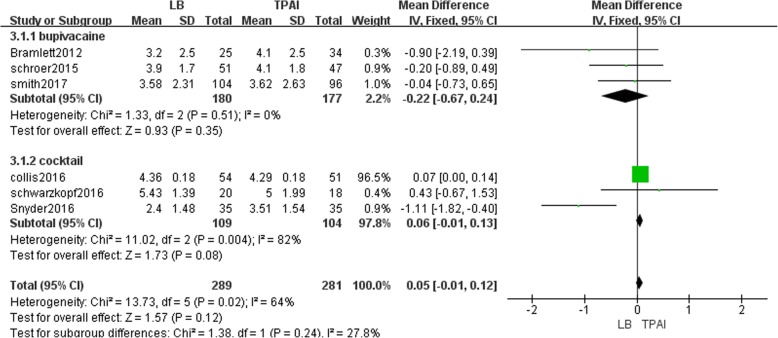
The frost plot of the VAS during the third 24 h after TKA

Six studies involving 570 patients recorded the VAS from 48 to 72 h postoperatively [12, 13, 18, 19, 23, 25]. The result showed that LB was not significantly different from TPAI regarding VAS during the third 24-h period after TKA (MD = 0.05, 95% CI: [− 0.01, 0.12], P = 0.12, I^2^ = 64%, Fig. 6). Fixed-effects meta-analysis revealed that the pain score in the liposomal bupivacaine group is not significantly reduced compared with the bupivacaine group (MD = -0.22, 95% CI: [− 0.67, 0.24], *P* = 0.35, I^2^ = 0%, Fig. 6), and not significantly increased compared with the cocktail group (MD = 0.06, 95% CI: [− 0.01, 0.13], *P* = 0.08, I^2^ = 82%, Fig. 6).

### Secondary outcome

#### The consumption of morphine equivalents during hospital stay

**Fig. 7:**
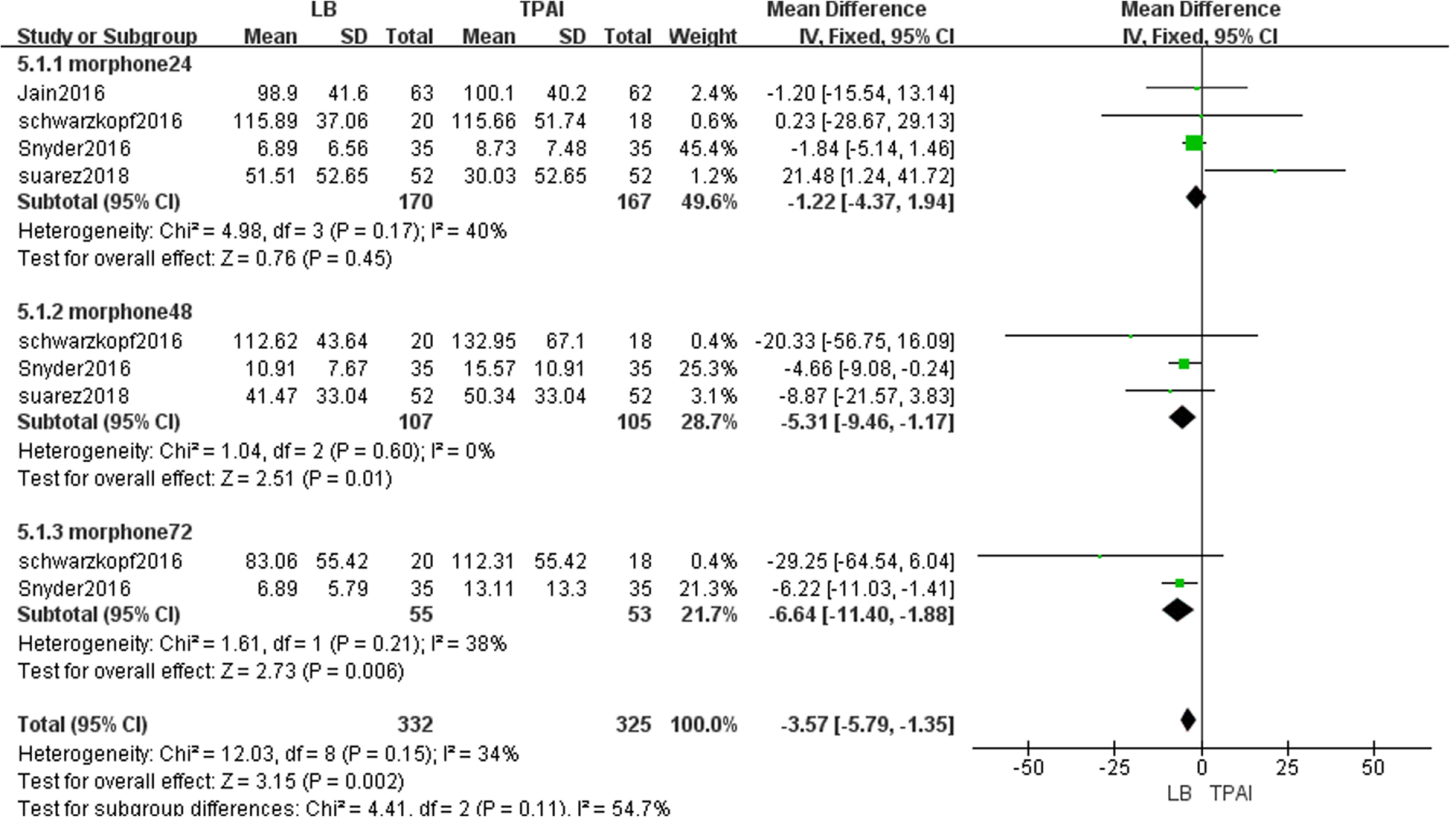
The frost plot of consumption of the morphine equivalents during the hospital stay

Four studies involving 337 patients reported the consumption of morphine equivalents from 0 to 72 h postoperatively [12, 21, 25, 26]. During the first 24 h, fixed-effects meta-analysis revealed that patients in the liposomal bupivacaine group did not consume significantly less morphine equivalents than TPAI (MD = -1.22, 95% CI: [− 4.37, 1.94], *P* = 0.45, I^2^ = 40%, Fig. 7). During the second 24-h period, fixed-effects meta-analysis revealed that patients in the liposomal bupivacaine group consumed significantly less morphine equivalents than TPAI (MD = -5.31, 95% CI: [− 9.46, − 1.17], *P* = 0.01, I^2^ = 0%, Fig. 7). During the third 24-h period, fixed-effects meta-analysis revealed that patients in the liposomal bupivacaine group consumed significantly less morphine equivalents than TPAI (MD = -6.64, 95% CI: [− 11.40, − 1.88], *P* = 0.006, I^2^ = 38%, Fig. 7).

#### The incidence of postoperative nausea and vomiting

**Fig. 8:**
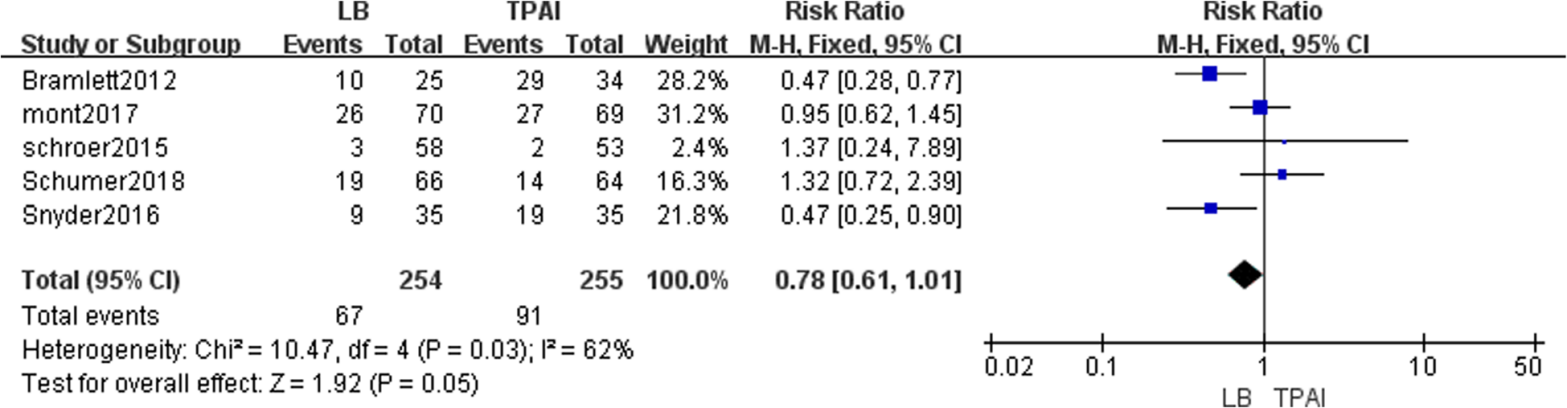
The frost plot of the incidence of nausea and vomiting after the TKA

Five studies involving 509 patients described the incidence of postoperative nausea and vomiting (NAVO) [18, 22–25]. Fixed-effects meta-analysis revealed that the incidence of NAVO in the liposomal bupivacaine group is reduced compared with the control group (RR = 0.78, 95% CI: [0.61, 1.01], *P* = 0.05, I^2^ = 62%, Fig. 8) A significant difference might be obtained from a larger sample size.

#### The length of hospital stay

**Fig. 9:**
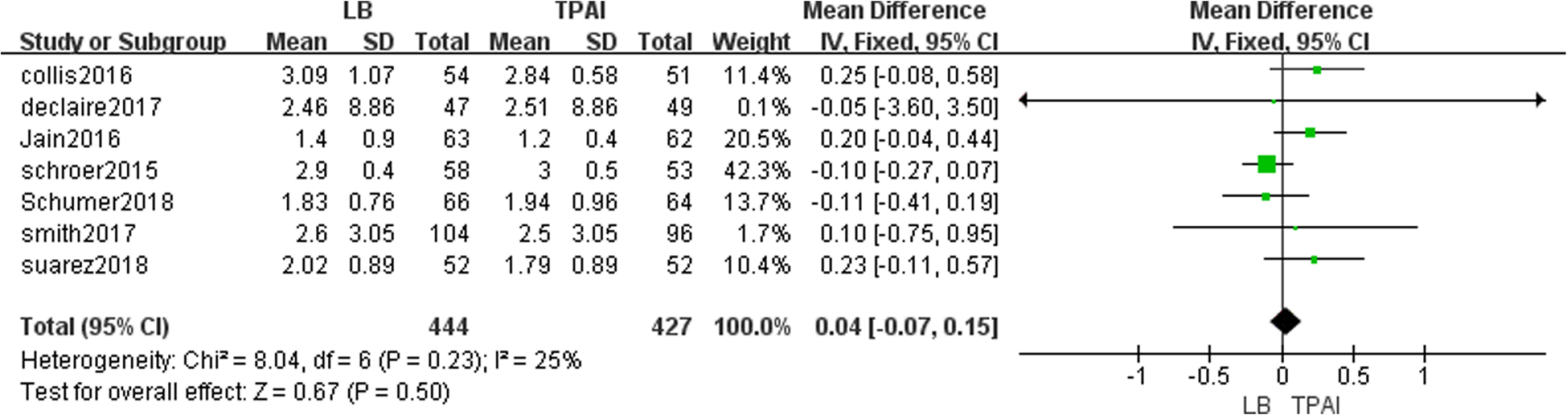
The frost plot of the length of hospital stay

Seven studies involving 871 patients recorded the length of hospital stay [13, 19–21, 23, 24, 26]. Fixed-effects meta-analysis revealed that the length of hospital stay in the liposomal bupivacaine group is not significantly longer compared with the control group (MD = 0.04, 95% CI: [− 0.07, 0.15], *P* = 0.50, I^2^ = 25%, Fig. 9).

### Quality of the evidence in the GRADE system

As shown in Table 2, a total of eleven outcomes in this meta-analysis were evaluated using the GRADE system (Table 2). The quality of evidence in the following two outcomes was high: VAS (0–24 h) cocktail and, length of stay. The outcome VAS (48–72 h) cocktail had a low quality of evidence. The remaining eight outcomes had moderate quality of evidence. Therefore, we believed that the overall evidence quality of our meta-analysis was very moderate.

**Table 2:**
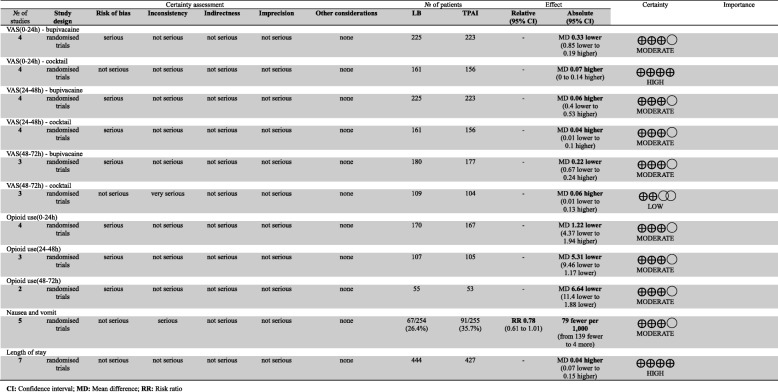
The GRADE evidence quality for each outcome

*CI* Confidence interval, *MD* Mean difference, *RR* Risk ratio

## Discussion

### Summary of findings

The purpose of our meta-analysis to explore the true efficacy of liposomal bupivacaine compared with traditional peri-articular injection following TKA. To our knowledge, this is the first meta-analysis that included more than 10 RCTs comparing LB and TPAI. The most significant finding of this study was that the liposomal bupivacaine did not make a difference regarding the visual analogue scale compared with traditional peri-articular injection after total knee arthroplasty. In other words, liposomal bupivacaine did not decrease the visual analogue scale compared with traditional peri-articular injection, which is the primary outcome of our review.

The primary outcome in our study is the postoperative VAS, which is the most intuitive indicator reflecting the efficacy of postoperative pain control. The final result revealed that liposomal bupivacaine did not show superiority regarding VAS during every 24-h period after TKA compared with TPAI, which is consistent with the result of a meta-analysis performed by Kuang et al. [10]. However, regarding the different amounts of additional top-up analgesia, we think the VAS scores do not reflect LB or bupivacaine alone. Therefore, we analysed the amount of opioid use after TKA as a secondary outcome to further compare the true efficacy of liposomal bupivacaine with control group.

The secondary outcome includes three items. First, the patients in the liposomal bupivacaine group consumed significantly less morphine equivalents from 24 to 72 h postoperatively, which revealed that liposomal bupivacaine has better efficacy of pain control after 24 h compared with TPAI. Consistently, pharmacokinetic data of liposomal bupivacaine exhibited bimodal kinetics with rapid uptake observed during the first few hours and prolonged release through 96 h after administration. [29]. Dasta et al. [6] reported that liposomal bupivacaine administered with the PAI technique was associated with statistically significant and clinically meaningful lower VAS score at 72 h compared with the control group. In addition, a review [30] concluded that liposomal bupivacaine might have a promising future as an extended release bupivacaine formulation with an approximately 72-h duration. Second, consistent with the reduced consumption of morphine equivalents, patients in the LB group have a reduced incidence of nausea and vomiting (NAVO) after TKA. Third, the length of the hospital stay was not significantly different between the two groups, suggesting that the liposomal bupivacaine group did not spend more time in functional recovery compared with the TPAI group.

### Strengths and limitations of the review

The first strength of our review was that we only include randomized controlled trials into our review, which improved our review’s level of evidence. In addition, more RCTs were included in our study than other reviews, which could help us acquire more believable results.

There are several limitations in our study. First, several actual analyses we report on only include a small sample of the 13 RCTs. For example, only 4 studies analysed opioid use, and 6 studies assessed VAS at varying time points. If more relevant, high-quality RCTs were included in our review, more useful information and results would be acquired. Second, the cocktail composition varied between studies and might influence the postoperative pain score and produce confounding bias. Third, given the limited number of included studies and data extracted, we did not compare the functional recovery between the two groups. However, the length of hospital stay was not significantly different in the two groups, revealing that the two groups spent a similar amount of time to reach the standard of leaving the hospital.

### Meaning of the study: implications for clinicians and researchers

Postoperative pain management plays an important role in guaranteeing the final successful outcome of TKA. Inadequate pain control after TKA increases the consumption of rescue opioids, disrupts sleep, influences physical exercise, increases the length of hospital stay and total cost, and decrease the degree of satisfaction [4, 31]. Greater than 700,000 TKA procedures performed annually in the USA [20], there is an urgent necessity to identify effective perioperative pain management that allows surgeons to be satisfied with the final outcome of TKA. Among the various types of measures, traditional peri-articular injection (TPAI) has been confirmed safe and effective in postoperative pain management after TKA [32–34]. Bupivacaine, ropivacaine, ketorolac, morphine, and epinephrine are usually used in combination as representative agents in a cocktail in TPAI.

Liposomal bupivacaine is an updated medication from bupivacaine, which is scraped from liposomes and injected into surgical site to alleviate postoperative pain. Since the drug was approved by the FDA in 2013, a series of clinical trials [35–37] and meta-analysis [9–11] have compared the efficacy of liposomal bupivacaine with traditional peri-articular injection (TPAI) following total joint arthroplasty (TJA). Lonner et al. [35] concluded that it is an “effective mechanism to assist in early hospital discharge and rapid recovery after TJA.” Barrington et al. [36] conduct a clinical trial with more than 1000 patients and demonstrated that LB showed superiority in lower pain scores and reduced length of hospital stay. Bagsby et al. [37] perform a retrospective cohort study and concluded that “liposomal bupivacaine PAI provided inferior pain control compared to the less expensive traditional PAI in a multi-modal pain control programme in patients undergoing TKA”. Interestingly, the efficacy of liposomal bupivacaine was better in the meta-analysis performed by Wang et al. [9], similar in the meta-analysis conducted by Kuang et al. [10], and worse in the meta-analysis performed by Sun et al. [11]. Obviously, the comparison has not reached a unified recommendation.

### Future directions

In terms of optimal efficacy and low cost, future studies should pay attention to the selection of the most effective drug composition for peri-articular injection for pain control after TKA. For example, Chai et al. [38] perform a meta-analysis and concluded that adding the corticosteroid to the multimodal cocktail is beneficial for pain control after TKA. Comparing the efficacy of liposomal bupivacaine with other analgesic methods is encouraged when three key points were considered. The first point is the consistency of the technique between the experiment and control group. We know liposomal bupivacaine was injected into the surgical site to reduce pain. Therefore, when we choose LB as the experimental group, use of the same peri-articular injection using different analgesic agents was ideal for the control group. This selection guarantees the consistency and decreases the confounding bias to yield credible outcomes in terms of statistics. The second point is that the time interval of 24 to 72 h postoperatively should receive more attention in the comparison. One advantage of liposomal bupivacaine is that it is effective to 72 h than general agents [30]. Therefore, attention should be paid to the comparison from 24 to 72 h postoperatively when we compared LB with other analgesic methods. Moreover, most patients left the hospital before 72 h postoperatively; thus, future studies should record the number of patients compared after 24 h correctly [39]. The third point is that combined analgesia should be taken into account. A series of studies compared adductor canal block (ACB) combined with liposomal bupivacaine versus femoral nerve block (FNB) or ACB combined with local injection versus local injection alone after TKA [40–42]. It can be inferred that combined analgesia would have better efficacy than the single method, but the increased cost of pain control had to be considered.

## Conclusion

Liposomal bupivacaine did not have an effect regarding the visual analogue scale compared with traditional peri-articular injection after total knee arthroplasty. However, liposomal bupivacaine was preferred in terms of reduced consumption of morphine equivalents during 24–72 h postoperatively and reduced incidence of nausea and vomiting after total knee arthroplasty.

## Data Availability

All data and materials are contained within the manuscript.

## Abbreviations

CI: Confidence interval
LB: Liposomal bupivacaine
LOS: Length of hospital stay
MD: Mean differences
NAVO: Nausea and vomiting
RCT: Randomized controlled trials
RR: Relative risks
TKA: Total knee arthroplasty
TPAI: Traditional peri-articular injection
VAS: Visual analogue score
